# The “life-span” of lytic polysaccharide monooxygenases (LPMOs) correlates to the number of turnovers in the reductant peroxidase reaction

**DOI:** 10.1016/j.jbc.2023.105094

**Published:** 2023-07-26

**Authors:** Silja Kuusk, Vincent G.H. Eijsink, Priit Väljamäe

**Affiliations:** 1 Institute of Molecular and Cell Biology, University of Tartu, Tartu, Estonia; 2 Faculty of Chemistry, Biotechnology and Food Science, NMBU - Norwegian University of Life Sciences, Ås, Norway

**Keywords:** lytic polysaccharide monooxygenase, peroxygenase, peroxidase, inactivation, hydrogen peroxide

## Abstract

Lytic polysaccharide monooxygenases (LPMOs) are monocopper enzymes that degrade the insoluble crystalline polysaccharides cellulose and chitin. Besides the H_2_O_2_ cosubstrate, the cleavage of glycosidic bonds by LPMOs depends on the presence of a reductant needed to bring the enzyme into its reduced, catalytically active Cu(I) state. Reduced LPMOs that are not bound to substrate catalyze reductant peroxidase reactions, which may lead to oxidative damage and irreversible inactivation of the enzyme. However, the kinetics of this reaction remain largely unknown, as do possible variations between LPMOs belonging to different families. Here, we describe the kinetic characterization of two fungal family AA9 LPMOs, *Tr*AA9A of *Trichoderma reesei* and *Nc*AA9C of *Neurospora crassa*, and two bacterial AA10 LPMOs, *Sc*AA10C of *Streptomyces coelicolor* and *Sm*AA10A of *Serratia marcescens*. We found peroxidation of ascorbic acid and methyl-hydroquinone resulted in the same probability of LPMO inactivation (*p*_i_), suggesting that inactivation is independent of the nature of the reductant. We showed the fungal enzymes were clearly more resistant toward inactivation, having *p*_i_ values of less than 0.01, whereas the *p*_i_ for *Sm*AA10A was an order of magnitude higher. However, the fungal enzymes also showed higher catalytic efficiencies (*k*_cat_/*K*_M(H2O2)_) for the reductant peroxidase reaction. This inverse linear correlation between the *k*_cat_/*K*_M(H2O2)_ and *p*_i_ suggests that, although having different life spans in terms of the number of turnovers in the reductant peroxidase reaction, LPMOs that are not bound to substrates have similar half-lives. These findings have not only potential biological but also industrial implications.

Lytic polysaccharide monooxygenases (LPMOs) are monocopper enzymes that catalyze the cleavage of glycosidic bonds in various polysaccharides and oligosaccharides. The most noteworthy property of LPMOs is their ability to break glycosidic bonds in recalcitrant, highly crystalline regions of insoluble substrates—cellulose and chitin. This can be achieved because of the flat and open active site architecture of LPMOs that is suited to interact with multiple polysaccharide chains in an ordered crystalline lattice (1, 2, 3, 4). The catalytically essential copper atom is held in a solvent exposed histidine-brace like structure that is part of a flat substrate-binding surface (5, 6, 7). This enables LPMOs to catalyze breakage of glycosidic bonds in polysaccharides while being in a regular crystal lattice. Thus, LPMO action does not depend on the energetically unfavorable lifting of the polysaccharide chain out of this lattice, which contrasts with canonical glycoside hydrolases, that act on single polysaccharide chains employing acid–base catalysis (8). LPMOs work synergistically with glycoside hydrolases and boost the rate of the degradation of recalcitrant polysaccharides (9, 10, 11, 12, 13, 14, 15, 16, 17).

Although initially described as monooxygenases, in 2017 Bissaro *et. al*., showed that LPMOs use H_2_O_2_ as a cosubstrate (18). Since then, several studies have confirmed the peroxygenase nature of LPMO catalysis (19, 20, 21, 22, 23, 24, 25, 26, 27, 28, 29) while the existence of a true monooxygenase activity is debated. Confusion regarding the nature of the cosubstrate stems from the fact that both peroxygenase and monooxygenase reactions rely on the Cu(I) form of the enzyme (30). Therefore, LPMOs need the presence of reductant that, for the peroxygenase activity, is used only for the initial priming of the Cu(II) resting state to the catalytically active Cu(I) form (18, 27, 31). Besides the initial priming reduction, the monooxygenase reaction requires stoichiometric delivery of two electrons per one glycosidic bond cleavage (9). Ascorbate (AscA) is the reductant most often used in LPMO research. Unfortunately, AscA is amenable to enzyme-independent oxidation by O_2_ and the product of such oxidation is H_2_O_2_, the true cosubstrate of LPMOs. Furthermore, the oxidation of AscA by O_2_ is catalyzed by copper (32, 33, 34), which is a plausible contaminant in LPMO reactions–it may be attached to sugar substrates or be present in LPMO preparations (22, 23, 35, 36). The situation is further complicated by the reductant oxidase activity of LPMOs. When not protected by the bound substrate, the Cu(I) active site of LPMOs can be reoxidized by O_2_ leading, again, to the formation of H_2_O_2_ (37).

Today, it is becoming widely accepted that the apparent monooxygenase activity in many LPMO reactions is a consequence of the H_2_O_2_-producing side reactions. The absence, or at least the lack of kinetic relevance, of the monooxygenase reaction is reflected in the low activity of LPMOs under typical “monooxygenase” experiment setups (38) and the strong stimulation of LPMO activity by factors stimulating the rate of H_2_O_2_-producing side reactions, like irradiation of light-sensitive redox-active compounds with visible light (39, 40, 41, 42, 43, 44, 45). Regarding interpretation of kinetic data, a serious drawback of LPMO studies performed with “monooxygenase” experimental setups is that the catalytic rates are limited by LPMO independent H_2_O_2_-producing side reactions (23, 46, 47, 48, 49) and, thus, do not reveal the true catalytic ability of the LPMO of interest. At best, catalytic rates obtained with these setups may reflect the reductant oxidase activity of the substrate-free LPMO under the given substrate load.

Similar to the oxidase activity described above, the Cu(I) active site of LPMOs can also be reoxidized by H_2_O_2_ in a reductant peroxidase reaction (24, 50, 51). The results of single-turnover measurements with AA9 and AA10 LPMOs have shown that the re-oxidation of Cu(I) by H_2_O_2_ is several orders of magnitude faster than re-oxidation by O_2_ (52, 53). An unwanted side reaction of reductant peroxidase activity is the irreversible inactivation of the enzyme. As proposed by Bissaro *et al*., in 2017, the polysaccharide peroxygenase activity involves Fenton-type chemistry (18), that is, homolytic cleavage of H_2_O_2_ (53), which generates a hydroxyl radical. Within the enzyme-substrate complex, the highly reactive hydroxyl radical intermediate is optimally positioned for productive chemistry, leading to hydrogen atom abstraction from the C1 and/or C4 carbon of the substrate (18, 54). However, in the absence of substrate the hydroxyl radical will engage in nonproductive reactions, such as oxidation of the enzyme, which may lead to the loss of catalytic activity. It has been shown that amino acids close to the catalytic copper are primary targets of oxidative damage (18, 55, 56).

Although structurally and biochemically well characterized, kinetic studies of peroxygenase catalysis by LPMOs are still scarce. To date only two in-depth kinetic studies of LPMOs acting on insoluble substrates are available, for the bacterial chitin-active family AA10 LPMO of *Serratia marcescens* (*Sm*AA10A) (19), and for the fungal family AA9 LPMO of *Trichoderma reesei* (*Tr*AA9A) (24). Kinetic characterization with soluble oligosaccharides is available for AA9 LPMOs of *Neurospora crassa* (*Nc*AA9C) (26) and *Lentinus similis* (*Ls*AA9A) (26, 28), and an AA11 of *Aspergillus fumigatus* (*Af*AA11B) (25). The kinetics of the reductant peroxidase reaction of LPMOs is also poorly characterized, as is the rate of enzyme inactivation associated with this reaction. In-depth kinetic characterization of the inactivation of LPMO through peroxidase reactions has only been described for one LPMO, *Tr*AA9A (24). The stability of LPMOs is of utmost importance for their application in biotechnological valorization of lignocellulosic biomass (57). However, the scarcity of kinetic data does not allow to conclude about possible activity-stability trade-offs.

To fill these knowledge gaps regarding the kinetic properties and stability of LPMOs, here, we provide the first kinetic characterization of the cellulose peroxygenase activity of a bacterial LPMO, *Sc*AA10C of *Streptomyces coelicolor*. We also provide in-depth kinetic characterization of the AscA peroxidase activity of this enzyme and three additional well-studied LPMOs, fungal cellulose-active *Tr*AA9A and *Nc*AA9C, and bacterial chitin-active *Sm*AA10A.

## Results

### Cellulose peroxygenase reaction

**Figure 1:**
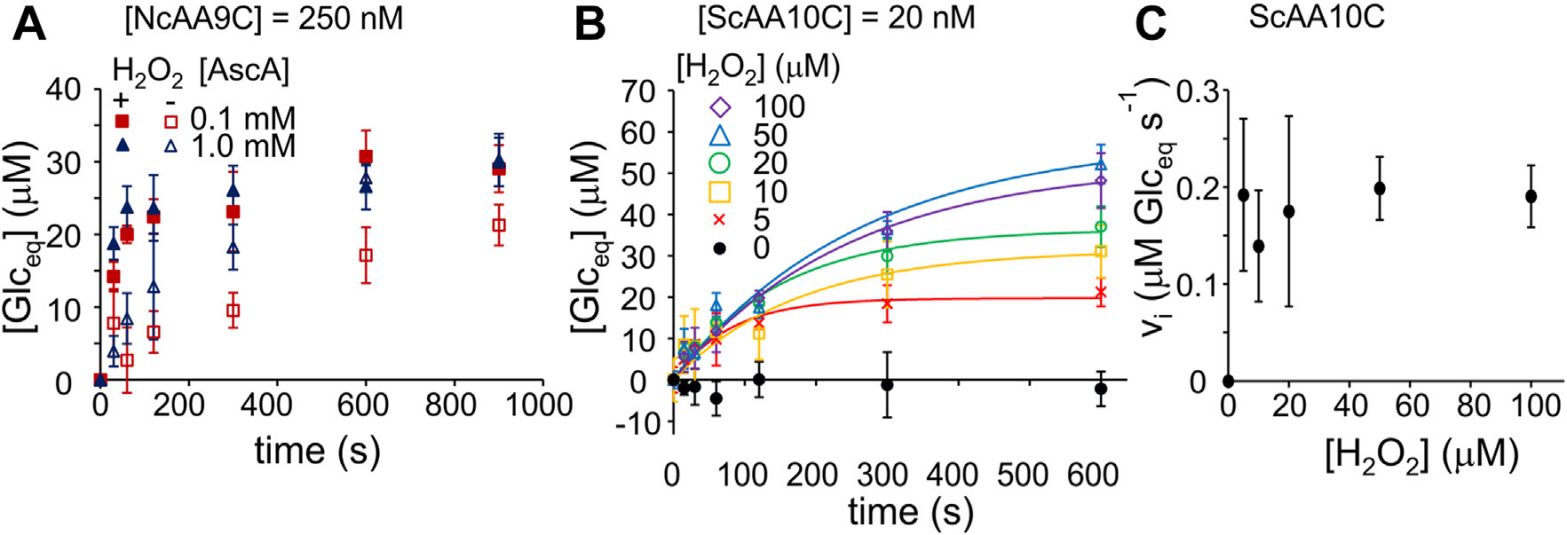
**Kinetics of the cellulose peroxygenase reaction**. All reactions were made in sodium acetate (50 mM, pH 5.0) at 25 °C. *A*, progress curves for the release of soluble products (expressed in glucose equivalents, Glc_eq_) upon incubation of ^14^C-BMCC (1.0 g L^−1^) with *Nc*AA9C (0.25 μM) in the presence (+) or absence (−) of added H_2_O_2_ (50 μM). The concentration of AscA was 0.1 or 1.0 mM. *B*, progress curves for the release of soluble products upon incubation of ^14^C-BMCC (1.0 g L^−1^) with *Sc*AA10 C (0.02 μM) in the presence of 1.0 mM AscA. The concentration of H_2_O_2_ is indicated in the plot. *Solid lines* show nonlinear regression of the data according to Equation 1. For the progress curves made using 1.5 g L^−1^ BMCC, Fig. S1*A*. *C*, dependency of the initial rates of the release of the soluble products (in μM Glc_eq_ s^−1^) from ^14^C-BMCC on the concentration of H_2_O_2_. The concentration of AscA was 1.0 mM. Shown are average values ± SD (*n* = 6, independent experiments) from the experiments made using 1.0 and 1.5 g L^−1^ BMCC. AscA, ascorbate; BMCC, bacterial microcrystalline cellulose.

To date an in-depth kinetic characterization of the polysaccharide peroxygenase reaction is available only for two LPMOs. Using ^14^C-labeled polymeric substrates we have characterized the degradation of chitin by *Sm*AA10A (19) and bacterial microcrystalline cellulose (BMCC) by *Tr*AA9A (24). Here we carried out similar studies for *Nc*AA9C and *Sc*AA10C. We chose pH 5.0 for kinetic characterization of LPMOs since this is the optimal pH for glycoside hydrolases that operate in synergy with LPMOs in degradation of lignocellulose. *Nc*AA9C is special because it is active on several soluble glycans and even cello-oligomers (58, 59, 60, 61). Although *Nc*AA9C was able to release soluble products from ^14^C-labeled BMCC, product levels were low and enzyme activity decayed rapidly (Fig. 1*A*). Differently from *Tr*AA9A (24) and *Sc*AA10C (see below), *Nc*AA9C showed significant activity also in the experiments without added H_2_O_2_. This apparent “monooxygenase” activity also decayed rapidly and using 1.0 mM AscA as reductant the reactions with and without added H_2_O_2_ reached the same plateau value of the released soluble products (Fig. 1*A*). These results suggest that BMCC is not a good substrate for *Nc*AA9C because of inefficient binding.

*Sc*AA10C had high activity on BMCC. Using 1.0 mM AscA as reductant, the release of soluble products in the experiments without added H_2_O_2_ was insignificant (Fig. 1*B*; note that these are 10 min reactions; reported LPMO activity in reductant-driven reactions is typically based on multi-hour incubation times). The decay of the release of ^14^C-labeled soluble products (expressed in glucose equivalents, Glc_eq_) in time was too fast to capture the linear-range of the progress curves (Figs. 1*B* and S1*A*). Therefore, the progress curves were fitted to a single exponential function (Equation 1) and initial rates were calculated as *v*_i_ = [*Glc*_*eq*_]_max_*k*_obs_ (time derivative of Equation 1 in the limiting conditions of time approaching to zero).

(1) $$\left[{Glc}_{eq}\right]={\left[{Glc}_{eq}\right]}_{\max}\left(1-e^{-k_{obs}t}\right)$$

The dependency of the initial rates of the release of soluble products on the concentration of [H_2_O_2_] is shown in Figure 1*C*. Unfortunately, the *K*_M_ for H_2_O_2_ appeared to be too low for determining its value. Within the error limits the activity was saturated with H_2_O_2_ already at the lowest concentration of H_2_O_2_ applicable (5.0 μM). The rates measured using 1.0 and 1.5 g L^−1^ BMCC were also the same within error limits, suggesting that the concentration of cellulose was saturating. Using the average values of initial rates measured using 5.0 to 100 μM H_2_O_2_ and 1.0 and 1.5 g L^−1^ BMCC we found *V*_max_ to be 0.18 ± 0.03 μM Glc_eq_ s^−1^. In order to convert the rates measured in Glc_eq_ s^−1^ to the turnover number for glycosidic bond cleavage, a stoichiometry coefficient (*n*) showing the number of soluble Glc_eq_ released per one glycosidic bond cleavage must be known. The value of *n* is measured under experimental conditions that favor the cellulose peroxygenase reaction, that is, at low H_2_O_2_ and high cellulose concentrations (24). Under such conditions, stoichiometric conversion of added H_2_O_2_ to cleaved glycosidic bonds can be assumed. A value of *n* = 3.7 ± 0.8 μM Glc_eq_/H_2_O_2_ was found using the average [*Glc*_*eq*_]_max_ (Fig. S1*B*) values obtained with 1.0 and 1.5 g L^−1^ BMCC, and 5.0 and 10 μM H_2_O_2_. We note that *n* is an empirical parameter that depends on the average degree of polymerization of the soluble products as well as on the probability of LPMO products being in the soluble fraction (19). However, *n* is useful for the purpose of calculating the *k*_cat_ of the cellulose peroxygenase reaction as it relies only on the assumption that the cleavage of a glycosidic bond depends on H_2_O_2_ and the stoichiometry is one glycosidic bond cleavage per one H_2_O_2_ molecule. Using an *n* of 3.7, the *k*_cat_ for the *Sc*AA10C-catalyzed cellulose peroxygenase reactions was calculated to be 2.4 ± 0.5 s^−1^.

Table 1 shows an overview of currently available kinetic parameters for the polysaccharide peroxygenase reaction catalyzed by LPMOs. The data show similar *k*_cat_ values for all three LPMOs (two AA10s and 1 AA9) but indicate that the AA10s have lower *K*_M_ values for H_2_O_2_.

**Table 1:** Kinetic parameters of the polysaccharide peroxygenase reaction

Enzyme	Substrate	*n*^a^	*k*_cat_	*K*_m(H2O2)_	*k*_cat_/*K*_m(H2O2)_
		(Eq/H_2_O_2_)	(s^−1^)	(μM)	(mM^−1^ s^−1^)
*Sm*AA10A^b^	^14^C-Chitin	4.0 ± 0.3	6.7 ± 0.2	2.8 ± 1.3	2400 ± 1100
*Tr*AA9A^c^	^14^C-BMCC	3.0 ± 0.15	8.5 ± 0.4	30 ± 5	290 ± 50
*Sc*AA10C	^14^C-BMCC	3.7 ± 0.8	2.4 ± 0.5	˂ 5	˃ 500

b From Kuusk *et al*. 2018 (measured at pH 6.1) (19).

c From Kuusk and Väljamäe 2021 (24).

a Stoichiometry coefficient (soluble monosaccharide equivalents per H_2_O_2_).

### Ascorbate peroxidase reaction

**Figure 2:**
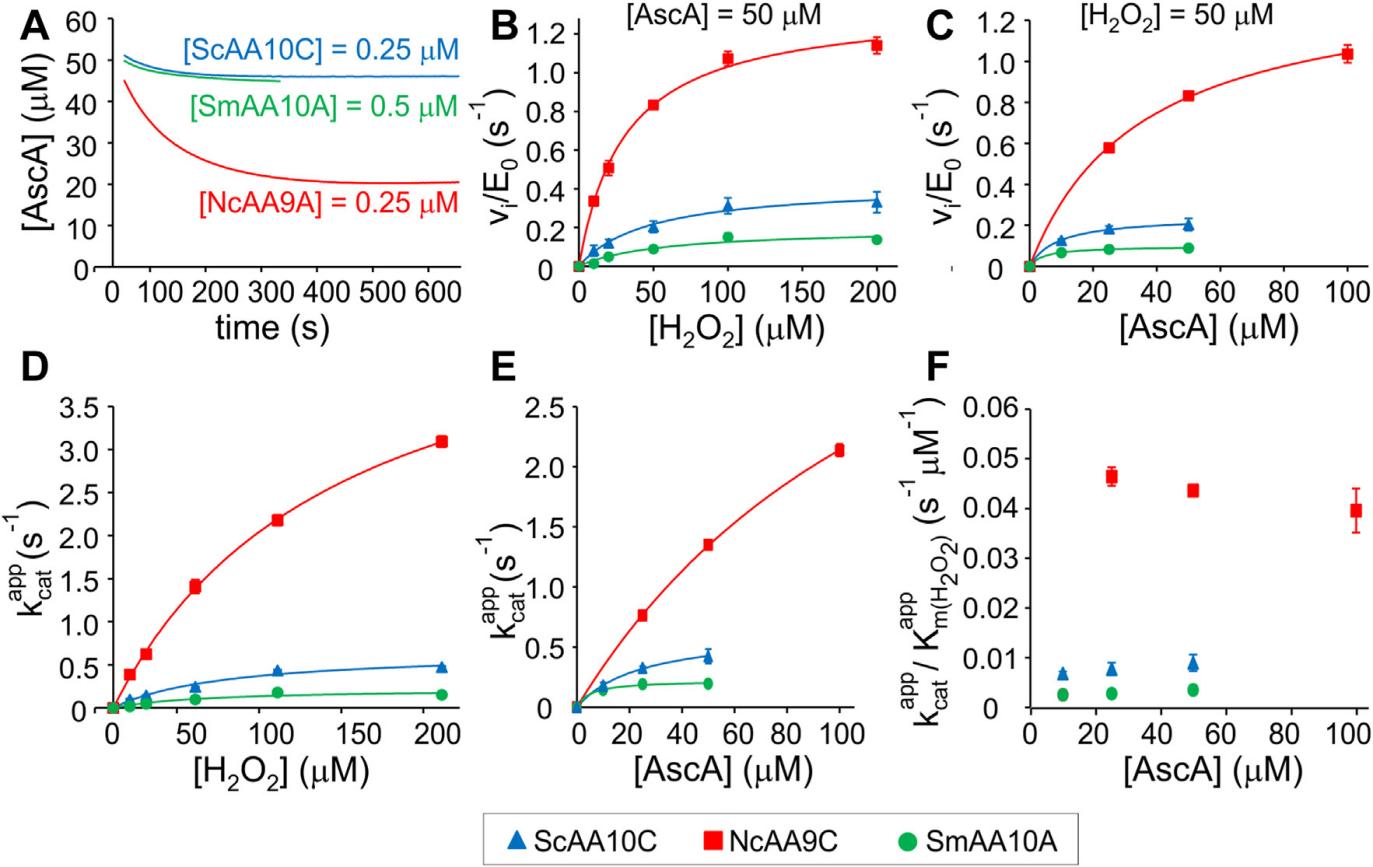
**Kinetics of the ascorbate peroxidase reaction.** All reactions were made in sodium acetate (50 mM, pH 5.0) at 25 °C. *A*, characteristic progress curves for the oxidation of AscA (50 μM). The concentrations of H_2_O_2_ was 100 μM and that of LPMOs is given on the plot. For progress curves for reactions with different [AscA] and [H_2_O_2_] Fig. S4. All progress curves were corrected for AscA oxidation in reactions without added H_2_O_2_ (Fig. S2). *B* and *C*, Michaelis–Menten curves for the oxidation of AscA by LPMOs. The substrate whose concentration was kept constant is indicated in the plots. *Solid lines* show nonlinear regression of the data according to the Equation 2. For Michaelis–Menten curves determined using different [AscA] and [H_2_O_2_] Fig. S5. *D* and *E*, the dependency of the apparent *k*_cat_ for the ascorbate peroxidation reaction on the concentration of H_2_O_2_ (*D*) or AscA (*E*). *Solid lines* show nonlinear regression of the data according to Equation 3. *F*, the dependency of the apparent *k*_cat_/*K*_M(H2O2)_ on the concentration of AscA. For the concentration dependency of the other parameters of the Micahelis–Menten equation, Fig. S6. Shown are average values ± SD (for *Sc*AA10C *n* = 3, and for the other LPMOs *n* = 2 independent experiments). For clarity SDs are not shown for the traces in (*A*). AscA, ascorbate; LPMOs, lytic polysaccharide monooxygenases.

AscA is the most often used reductant in LPMO research. However, to date the kinetic characterization of the AscA peroxidase reaction is available only for *Tr*AA9A (24). Here, we extended the studies of the AscA peroxidase reaction to three model LPMOs, *Nc*AA9C, *Sc*AA10C, and *Sm*AA10A. Characteristic progress curves for AscA oxidation are shown in Figure 2*A*. With all LPMOs tested, the rate of AscA oxidation decayed in time and approached zero within the measurement period of 10 min. There was also a slow oxidation of AscA in the experiments without added LPMO (nonenzymatic oxidation) or H_2_O_2_ (AscA oxidase activity of LPMO) (Fig. S2), and the progress curves reported in Figure 2 and below were always corrected for the rate of the oxidation of AscA in experiments without added H_2_O_2_, which, as shown in Fig. S3, was much lower than that measured in the experiments with added H_2_O_2_.

With both, [H_2_O_2_] (Figs. 2*B* and S5, *A*–*C*) and [AscA] (Figs. 2*C* and S5, *D* and *F*) as variable, the initial rates (normalized to the enzyme concentration, *v*_i_/*E*_0_) of AscA oxidation were consistent with Michaelis–Menten saturation kinetics (Equation 2).

(2) $$\frac{v_{i}}{E_{0}}=\frac{k_{cat}^{app}\left[S\right]}{K_{M\left(S\right)}^{app}\;+\;\left[S\right]}$$

In Equation 2 [S] stands for the concentration of the substrate, the concentration of which was varied within the series (H_2_O_2_ or AscA), and *k*_cat_^app^ and *K*_M(S)_^app^ are apparent catalytic and Michaelis constants, respectively.

Enzyme catalyzed reactions involving two substrates obey either the ternary complex or the ping-pong kinetic mechanism. The kinetic signature of the ping-pong mechanism is that the apparent *k*_cat_/*K*_M_ is always the true value independent of the concentration of the other substrate (62). This was shown to be the case for the AscA peroxidase reaction of *Tr*AA9A (24). It seems that, within the experimental scatter, there is no clear dependency of *k*_cat_^app^/*K*_M(H2O2)_^app^ on [AscA] (Fig. 2*F*) nor of *k*_cat_^app^/*K*_M(AscA)_^app^ on [H_2_O_2_] (Fig. S6*A*) for *Nc*AA9C, *Sc*AA10C, and *Sm*AA10A, suggesting that reductant peroxidation in these enzymes is best described by a ping-pong mechanism (for the dependency of *K*_M(H2O2)_^app^ on [AscA] and *K*_M(AscA)_^app^ on [H_2_O_2_] Fig. S6, *B* and *C*, respectively).

Since the dependency of *k*_cat_^app^ on the concentration of the substrate that was kept constant within the series is the same for both the ping-pong and the ternary complex mechanisms, the true values of kinetic parameters were derived from the dependency of *k*_cat_^app^ on [H_2_O_2_] (Fig. 2*D*) and [AscA] (Fig. 2*E*) according to Equation 3.

(3) $$k_{cat}^{app}=\frac{k_{cat}\left[S\right]}{K_{M\left(S\right)}\;+\;\left[S\right]}$$

In Equation 3 [S] stands for the concentration of the substrate, the concentration of which was kept constant within the series (H_2_O_2_ or AscA) and *K*_M(S)_ is the true Michaelis constant for the same substrate. The values of the true kinetic parameters for the AscA peroxidase reaction are listed in Table 2. Fungal AA9 enzymes showed higher *k*_cat_ and *k*_cat_/*K*_M(H2O2)_ values compared to their bacterial AA10 counterparts. On the other hand, AA10 enzymes tend to have higher apparent affinity for AscA (lower *K*_M(AscA)_). The differences between fungal and bacterial LPMOs in the terms of *K*_M(H2O2)_ and *k*_cat_/*K*_M(AscA)_ were less obvious (Table 2).

**Table 2:** Kinetic parameters of the ascorbate peroxidase reaction

Enzyme	*k*_cat_	*K*_m(H2O2)_	*K*_m(AscA)_	*k*_cat_/*K*_m(H2O2)_	*k*_cat_/*K*_m(AscA)_
	(s^−1^)	(μM)	(μM)	(mM^−1^ s^−1^)	(mM^−1^ s^−1^)
*Tr*AA9A^a^	2.1 ± 0.2	78	140 ± 25	27 ± 3	15 ± 3
*Nc*AA9C	5.3 ± 0.1^b^ 5.2 ± 0.2^c^	139 ± 7	145 ± 4	37 ± 2	36 ± 1
*Sc*AA10C	0.64 ± 0.02^b^ 0.66 ± 0.08^c^	70 ± 20	25 ± 2	9.4 ± 2.7	21 ± 2
*Sm*AA10A	0.22 ± 0.01^b^ 0.22 ± 0.05^c^	60 ± 34	5.2 ± 1.3	3.8 ± 2.2	43 ± 11

a From Kuusk and Väljamäe 2021 (24).

b Derived from the dependency of *k*_cat_^app^ on [AscA] (Fig. 2*E*).

c Derived from the dependency of *k*_cat_^app^ on [H_2_O_2_] (Fig. 2*D*).

### Inactivation of LPMOs

A drawback in LPMO catalysis is the irreversible inactivation of the enzyme in the reductant peroxidase reaction. For quantitative analysis of inactivation the time curves of AscA oxidation (Figs. 2*A* and S4) were analyzed according to the Equation 4 (24).

(4) $$\left[AscA\right]=\Delta {\left[AscA\right]}_{\max}e^{-k^{app}t}\;+\;{\left[AscA\right]}_{\infty }$$

**Figure 3:**
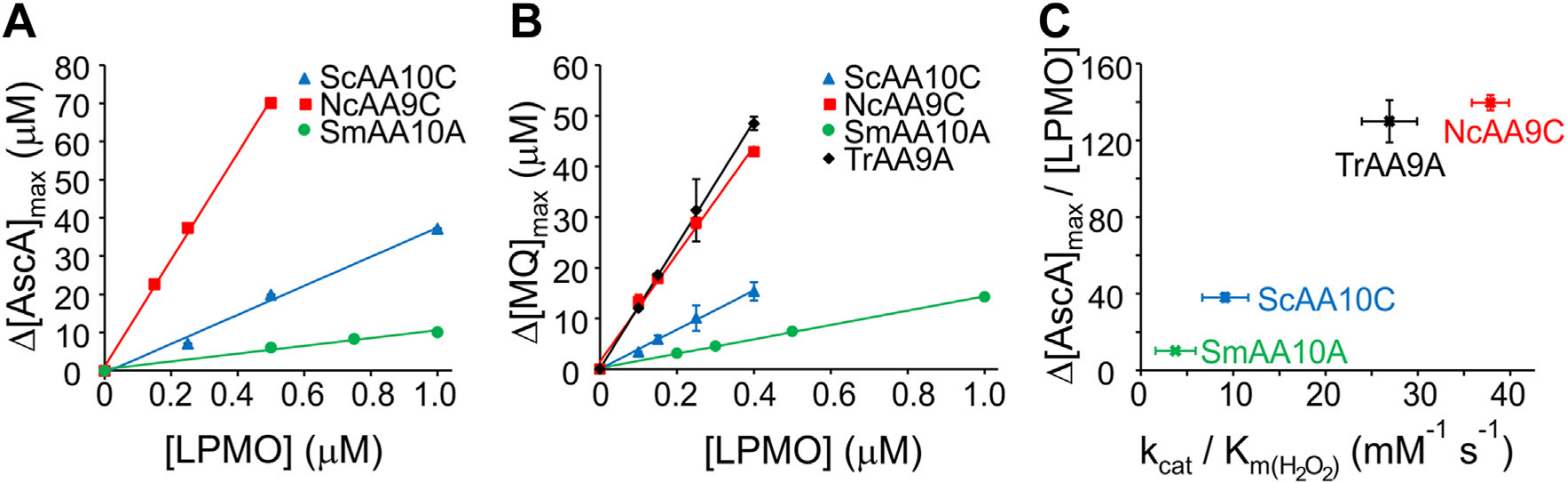
**Inactivation of LPMOs in the reductant peroxidase reaction.** All reactions were made in sodium acetate (50 mM, pH 5.0) at 25 °C. *A* and *B*, dependency of the maximum amount of ascorbate (*A*) or methyl hydroquinone (*B*) turned over on the concentration of the LPMO. *Solid lines* show the linear regression of the data. The slope of the line defines *n*_max_ (see Equation 5). *C*, correlation between *n*_max_ for AscA and *k*_cat_/*K*_M(H2O2)_ for the ascorbate peroxidase reaction. The data for *Tr*AA9A are from Ref (24). For the correlation between *n*_max_ for AscA and other parameters of the ascorbate peroxidase reaction Fig. S11. Shown are average values ± SD (*n* = 2, independent experiments). AscA, ascorbate; LPMOs, lytic polysaccharide monooxygenases.

In Equation 4, Δ[AscA]_max_ and [AscA]_∞_ are the maximum change and the remaining concentration of AscA, respectively, and *k*^app^ is the apparent first order rate constant for AscA oxidation. Importantly, in conditions where AscA and H_2_O_2_ are not limiting (not depleted) Δ[AscA]_max_ represents the maximum number of AscA molecules turned over before full inactivation of the LPMO. Indeed, Δ[AscA]_max_ values derived from progress curves for reactions with high AscA and H_2_O_2_ concentrations (Fig. S7) scaled linearly with the total concentration of LPMO (Fig. 3*A*). The slope of this line (Δ[AscA]_max_ *versus* [LPMO]) represents the average number of peroxidase reactions that one LPMO can catalyze before irreversible inactivation (*n*_max_).

(5) $$n_{max}=\frac{\Delta {\left[Reductant\right]}_{\max}}{\left[LPMO\right]}=\frac{1}{p_{i}}$$

The term *p*_i_ in Equation 5 stands for the probability of LPMO inactivation in the reductant peroxidase reaction. Using the data depicted in Figure 3*A* (with underlying progress curves shown in Fig. S7), we determined Δ[AscA]_max_ values and the *p*_i_ for the different LPMOs and the data are summarized in Table 3. Fungal enzymes had much higher stability, turning over more than 100 AscA molecules before inactivation, while *Sc*AA10A and *Sm*AA10A were inactivated after 38 and 10 turnovers, respectively.

**Table 3:** Inactivation of LPMOs in the reductant peroxidase reaction

Enzyme	Δ[AscA]_max_/[LPMO]^a^	Δ[MQ]_max_/[LPMO]^b^	*p*_i_^c^
*Tr*AA9A	130 ± 11^d^	122 ± 1	0.0079 ± 0.0007
*Nc*AA9C	140 ± 4	106 ± 2	0.0081 ± 0.0002
*Sc*AA10C	38 ± 2	39 ± 4	0.026 ± 0.0027
*Sm*AA10A	10 ± 1	14 ± 0.5	0.083 ± 0.0083

a Maximum number of AscA molecules turned over per one molecule of LPMO.

b Maximum number of MHQ molecules turned over per one molecule of LPMO.

c Probability of inactivation. Calculated according to the Equation 5 using average *n*_max_ values from the experiments with AscA and MHQ.

d From Kuusk and Väljamäe 2021 (24).

To verify these differences and exclude possible reductant-specific effects on LPMO inactivation, we also tested the peroxidation of a phenolic reductant, methyl hydroquinone (MHQ) by LPMOs. At high MHQ and H_2_O_2_ concentrations, the formation of the oxidized product (methyl quinone, MQ) decayed because of inactivation of LPMO, as shown for *Tr*AA9A in Fig. S8. We were not able to obtain stock solutions of MHQ without small amounts of H_2_O_2_, which precluded proper correction for background (O_2_-driven) MHQ turnover and Michaelis–Menten analysis. Therefore, the data from experiments without LPMO were used as the background signal (Fig. S8). Based on progress curves for the oxidation of MHQ by LPMOs (Fig. S9) we first selected suitable time points (180 min incubations for *Tr*AA9A and *Nc*AA9C, and 50 min and 30 min incubations for *Sc*AA10A, and *Sm*AA10A, respectively) and used single time point analysis for the determination of maximum amount of oxidized MHQ (Δ[MQ]_max_). Similar to what was seen with AscA, the Δ[MQ]_max_ scaled linearly with the concentration of LPMO (Fig. 3*B*). Most importantly, the peroxidation of two different reductants resulted in similar *n*_max_ (and, thus, *p*_i_) values (Table 3), suggesting that inactivation of LPMOs in the peroxidase reaction is independent of the nature of the reductant.

To verify that enzyme inactivation is general and not related to the peroxidase reaction only, we did preincubation experiments with *Tr*AA9A and *Sc*AA10C. After preincubation with H_2_O_2_ and the reductant (AscA and MHQ; peroxidase conditions) for selected times, we measured residual BMCC peroxygenase activity. In all cases, the loss of the activity in the reductant peroxidase reaction was reflected in the loss of activity in the cellulose peroxygenase reaction (Fig. S10), suggesting that both reactions are similarly affected by inactivation.

Comparison of the kinetic parameters for the AscA peroxidase reaction (Table 2) and the maximum turnover numbers (Table 3) revealed a positive correlation (increasing *n*_max_ with increasing parameter value) between *k*_cat_ (Fig. S11*A*), *k*_cat_/*K*_M(H2O2)_, (Fig. 3*C*), and *K*_M(AscA)_ (Fig. S11*B*) for the AscA peroxidase reaction and *n*_max_. No such correlation was found between *K*_M(H2O2)_ (Fig. S11*C*) or *k*_cat_/*K*_M(AscA)_ (Fig. S11*D*) and *n*_max_.

## Discussion

Recent studies have shown that LPMOs are efficient polysaccharide peroxygenases (19, 20, 21, 22, 23, 24, 25, 26, 27, 28, 29). For the catalysis of this unique reaction, these monocopper enzymes rely on a single Cu(I) in their active site (30). Since the resting state of copper in aerobic environments is Cu(II), LPMOs need the presence of a reductant for their activation. To date there is no evidence for the reoxidation of the Cu(I) LPMO in the polysaccharide peroxygenase reaction. On the contrary, multiple studies have shown that, once reduced, an LPMO can perform multiple peroxygenase reactions (18, 27, 31). Although it has been shown that the Cu(I) form of LPMO binds to the substrate with higher affinity compared to the Cu(II) form (31, 63), in real systems there is always a population of substrate-free LPMO-Cu(I). This population is amenable to reoxidation by O_2_ and H_2_O_2_. Since LPMOs acting on crystalline surfaces have evolved a flat, solvent-exposed active site architecture, off-pathway reoxidation of the active site copper may be an unavoidable side reaction. Reoxidation by O_2_ to generate H_2_O_2_ has been studied quite well and it has been speculated that the reductant oxidase activity of LPMO may serve as a source of H_2_O_2_ to be used by LPMOs (25, 49) or by other H_2_O_2_ consuming enzymes like lignin peroxidases (64, 65). On the other hand, less is known about reoxidation by H_2_O_2_, whereas this reaction actually is crucial because it may lead to the irreversible enzyme inactivation of LPMO. Of note, it is hard to see any biological rationale for the reductant peroxidase activity of LPMOs especially in the light of enzyme inactivation.

It is important to minimize the flux of H_2_O_2_ through the reductant peroxidase reaction, to maximize LPMO stability and minimize futile turnover of oxidant. Assuming the experimentally supported ping-pong mechanism, the rate of H_2_O_2_ turnover in the reductant peroxidase reaction (*v*_ox_^R^) is given by Equation 6.

(6) $$\frac{v_{ox}^{R}}{E_{0}}=\frac{k_{cat}^{R}}{1\;+\;\frac{K_{M\left(R\right)}^{R}}{\left[R\right]}\;+\;\frac{K_{M\left(H2O2\right)}^{R}}{\left[H_{2}O_{2}\right]}}$$

In Equation 6, R denotes the reductant, and the kinetic parameters of the reductant peroxidase reaction (Table 2) are designated with superscript R. H_2_O_2_ is a reactive molecule and its steady-state concentrations in biological environments are expected to be low. When applying the constraints of [H_2_O_2_] << *K*_M(H2O2)_^R^ and (*K*_M(H2O2)_^R^/[H_2_O_2_]) >> (*K*_M(R)_^R^/[R]) Equation 6 simplifies to Equation 7.

(7) $$\frac{v_{ox}^{R}}{E_{0}}\;\approx \;\frac{k_{cat}^{R}}{K_{M\left(H2O2\right)}^{R}}\left[H_{2}O_{2}\right]$$

Equation 7 shows that, under these assumptions, the rate of the reductant peroxidase reaction depends linearly on the H_2_O_2_ concentration. *k*_cat_^R^ and *K*_M(H2O2)_^R^ may vary between reductants and will, as shown in this study, vary between LPMOs (Table 2).

The LPMO peroxygenase reaction has been shown to follow the ternary complex mechanism (19, 24), the kinetics of which is given by Equation 8.

(8) $$\frac{v_{ox}^{S}}{E_{0}}=\frac{k_{cat}^{S}}{1\;+\;\frac{K_{M\left(S\right)}^{S}}{\left[S\right]}\;+\;\frac{K_{M\left(H2O2\right)}^{S}}{\left[H_{2}O_{2}\right]}\left(1\;+\;\frac{K_{i\left(S\right)}^{S}}{\left[S\right]}\right)}$$

In Equation 8, S denotes the sugar substrate, and the kinetic parameters for the peroxygenase reaction (Table 1) are designated with superscript S. The term *K*_i(S)_^S^ in Equation 8 stands for the binding constant of sugar substrate in the absence of H_2_O_2_. Within the constraints of low [H_2_O_2_] and high [S] Equation 8 simplifies to a form equivalent to Equation 7 derived for the reductant peroxidase reaction above. Thus, at low concentration of H_2_O_2_ and high concentration of cellulose, which both are plausible assumptions for the conditions in the natural environments of LPMOs, the relative contribution of H_2_O_2_ fluxes through the cellulose peroxygenase (*v*_ox_^S^) and reductant peroxidase reactions (*v*_ox_^R^) are given by Equation 9.

(9) $$\frac{v_{ox}^{S}}{v_{ox}^{R}}=\frac{{\left[LPMO\right]}_{bound}\left(k_{cat}^{S}/K_{M\left(H2O2\right)}^{S}\right)}{{\left[LPMO\right]}_{free}\left(k_{cat}^{R}/K_{M\left(H2O2\right)}^{R}\right)}$$

In Equation 9 [LPMO]_bound_ and [LPMO]_free_ stand for the concentrations of LPMO populations with the active site productively bound to the sugar substrate and free from the sugar substrate (*i.e.* available for the reductant peroxidase reaction), respectively. Comparison of the kinetic data in Tables 1 and 2 reveals that, in terms of *k*_cat_/*K*_M(H2O2)_ values, the flux of H_2_O_2_ through the peroxygenase reaction is favored only by a factor of 10 ± 2 for *Tr*AA9A, whereas the corresponding figure for *Sm*AA10A is 740 ± 430. Although the low *K*_M(H2O2)_ value (Fig. 1*C*) precluded determination of the *k*_cat_/*K*_M(H2O2)_ value of the cellulose peroxygenase reaction catalyzed by *Sc*AA10C, it seems, that also this bacterial enzyme has a strong preference, by a factor 50 or higher, in favor of the peroxygenase reaction (Tables 1 and 2).

As shown by Equation 9, the H_2_O_2_ fluxes depend on the concentrations of substrate-bound and free LPMO, which again will depend on the substrate concentration. Indeed, it has been shown in several studies that substrate affinity is an important contributor to LPMO stability (66). Stronger binding increases the [LPMO]_bound_/[LPMO]_free_ ratio and drives the flux of H_2_O_2_ through the peroxygenase reaction (Equation 9). The results obtained when characterizing the cellulose peroxygenase kinetics of *Nc*AA9C (Fig. 1*A*) may serve as an example of the effects of inefficient binding. Although the initial activity seems to be high, the enzyme is rapidly inactivated in the experiments with added H_2_O_2_. *Nc*AA9C had also relatively high initial activity (compared to *Sc*AA10C (Fig. 1*B*) and *Tr*AA9A (24)) in the experiments without added H_2_O_2_ but also in this case the enzyme was rapidly inactivated (Fig. 1*A*). All in all, these results indicate that the reaction with BMCC is substrate-limited and, thus, that BMCC is not a good substrate for *Nc*AA9C. Although *Nc*AA9C has a carbohydrate binding module, inefficient binding of the catalytic domain would leave the active site free for “self-production” of H_2_O_2_ in the AscA oxidase reaction but also for inactivation in the AscA peroxidase reaction. Collectively these data suggest that crystalline BMCC is not a good substrate for *Nc*AA9C. It is conceivable that the enzyme only acted on a minor fraction of more amorphous material in the BMCC, and the effective substrate concentration thus was very low. Of note, activity of this enzyme on crystalline cellulose (Avicel) (58, 67) has been demonstrated, but only under rather extreme conditions (high enzyme loads, long incubations, and sensitive detection without quantitative reporting), that are very different from the conditions used here. The lack of activity on crystalline substrates is intriguing, especially considering that *Nc*AA9C clearly is a competent LPMO when acting on other substrates. For example, a *k*_cat_ value of 124 ± 27 s^−1^ (at 4 °C) has been reported for H_2_O_2_ driven cleavage of soluble cellopentaose (26).

Although the *k*_cat_/*K*_M(H2O2)_ values of the fungal enzymes (Tables 1 and 2) were less supportive for the flux of H_2_O_2_ through the peroxygenase reaction, the fungal LPMOs were more resistant toward inactivation in the reductant peroxidase reaction. The probability of inactivation of *Sm*AA10A in the reductant peroxidase reaction is about an order of magnitude higher compared to *Tr*AA9A and *Nc*AA9C (Table 3). Thus, the less pronounced preference for the peroxygenase reaction in fungal LPMOs seems to be, at least to some extent, counterbalanced by a higher resistance toward oxidative inactivation in the peroxidase reaction. The strong positive correlation between the *k*_cat_/*K*_M(H2O2)_ of the reductant peroxidase reaction and the number of turnovers made before the inactivation (Fig. 3*C*) suggests that the higher stability of fungal LPMOs has coevolved with the catalytic efficiency in the peroxidase reaction. One may further speculate that the latter is an unavoidable “coproduct” of evolution toward higher reductant oxidase efficiency needed for being “self-supporting” with H_2_O_2_ cosubstrate.

A characteristic structural feature of natural AA9 enzymes expressed in fungi is the *N*-methylation of the Cu coordinating N-terminal histidine (35). Studies of H_2_O_2_-fueled LPMO reactions have led to the suggestion that this posttranslational modification helps protect against oxidative damage to this vital residue (68). Importantly, of the two AA9s used in this study, only one, *Tr*AA9A, carried the methylation, whereas the two enzymes showed almost identical susceptibilities to inactivation through the peroxidase reaction (Table 3). LPMOs show large functional differences also within the same family, (69) and the similar stability of *Nc*AA9C and *Tr*AA9A does not rule out an important role of the methylation. However, the present results show that other structural features also play important roles. Another difference between fungal and bacterial LPMOs is that the fungal enzymes have a Tyr in the second coordination sphere located close to what could be called the proximal axial coordination position of the copper, whereas the corresponding position in about 90 % of bacterial AA10 enzymes, including the two studied here, is occupied by Phe (3, 70). Tyr and Trp have been proposed to protect redox enzymes against oxidative damage by providing hole hopping pathways for reactive radical intermediates (71). The presence of Trp and Tyr radical intermediates in the reoxidation of Cu(I) has indeed been demonstrated for fungal LPMOs, including *Tr*AA9A (53). Thus, the Tyr (instead of Phe) in the second coordination sphere may contribute to the higher stability of fungal LPMOs compared to their bacterial AA10 counterparts.

The results presented here suggest that the probability of the inactivation of LPMO in the reductant peroxidase reaction is independent on the nature of the reductant (Fig. 3, *A* and *B* and Table 3). This is expected for the ping-pong mechanism, where there is no ternary complex comprised of the LPMO, the reductant, and H_2_O_2_. Given that the inactivation takes place in the reaction of reduced Cu(I) LPMO with H_2_O_2_, and not in the reaction with Cu(II) LPMO, it is not surprising that it does not matter which specific reductant was responsible for generating the Cu(I) LPMO.

The linear correlation between the *k*_cat_/*K*_M(H2O2)_ for the reductant peroxidase reaction and the stability of the LPMO (Fig. 3*C*) reveals an interesting phenomenon of LPMO catalysis in low [H_2_O_2_] conditions. The rate constant of the inactivation of LPMO is a product of the apparent first order rate constant of the reductant peroxidase reaction (*k*_cat_^R^[H_2_O_2_]/*K*_M(H2O2)_^R^, Equation 7) and the probability of inactivation (*p*_i_, Equation 5). Thus, the half-life (*t*_1/2_) of the LPMO population that is not bound to substrate is given by Equation 10.

(10) $$t_{\left(1/2\right)}=\frac{\ln\;2}{\frac{k_{cat}^{R}}{K_{M\left(H2O2\right)}^{R}}\left[H_{2}O_{2}\right]p_{i}}$$

The linear decrease of *p*_i_ (compare increase of *n*_max_, Equation 5) with the increasing *k*_cat_^R^/*K*_M(H2O2)_^R^ (Fig. 3*C*) suggests that, although having very different life-spans in terms of the number of H_2_O_2_ turnovers in the peroxidase reaction, different LPMOs have similar half-lives in units of time. This half-life increases linearly with decreasing steady-state H_2_O_2_ concentration, (see Equation 10) but considering the ping-pong mechanism is expected to be independent of the concentration of the reductant (*i.e.*, the apparent *k*_cat_^R^/*K*_M(H2O2)_^R^ is independent on [R], Fig. 2*F*).

Although not the focus of this study, it is worth contemplating on the possible impact of the findings and considerations described above on the processing of cellulosic biomass with LPMO-containing enzyme cocktails (72). As degradation reactions proceed, the effective substrate concentration decreases, whereas the degradation of the remaining substrate, which likely is the most recalcitrant fraction of the starting material, would benefit from LPMO action. Instead, as a result of the lower substrate concentration, a larger fraction of the LPMOs will be in a substrate-free form, which leads to increased nonproductive use of H_2_O_2_ and increased enzyme inactivation. It is conceivable that process optimization could be achieved by nonconventional dosing of the LPMOs, rather than adding the LPMOs all at the start of the reaction. Further understanding and optimization of LPMO performance would benefit from more in-depth knowledge of substrate binding kinetics and of possible protective mechanisms (27, 55, 71) that may be affected by substrate binding.

Back to biology, one may wonder whether the different kinetic signatures of fungal and bacterial LPMOs reported here may reflect adaptation to different steady-state levels of H_2_O_2_ and/or the nature of the reductants present in their native environments. As recently discussed by Hemsworth (73), more detailed information about the conditions in the natural environments of LPMOs is needed to understand LPMO functionality in natural ecosystems and to reveal the biological relevance of functional differences described above.

## Experimental procedures

### Materials

MHQ (lot # BCBH9920V) and L-ascorbic acid (AscA, lot # SLBM0850V) were from Sigma-Aldrich. Chelex 100 resin (50–100 mesh, sodium form) was from Bio-Rad. The H_2_O_2_ stock solution (lot # SZBG2070) was from Honeywell. A 0.5 M stock solution of the sodium acetate buffer, pH 5.0, was kept overnight with beads of Chelex 100 resin after preparation. Dilutions of the commercial H_2_O_2_ stock solution (30 wt %, 9.8 M) were prepared in Chelex-treated sodium acetate buffer directly before use. AscA (50 mM in water) was kept as frozen aliquots at −18 °C and the aliquots were melted directly before use. The water was Milli-Q ultrapure water that had been passed through a column with Chelex 100 resin.

*Tr*AA9A, *Nc*AA9C, *Sc*AA10C, and *Sm*AA10A were produced and purified as described in Kont *et al*. (46), Kittl *et al*. (37), Forsberg *et al*. (74) and Vaaje-Kolstad *et al*. (1), respectively. The purified LPMOs were saturated with copper by overnight incubation with excess (3:1 M ratio) CuSO_4_. The unbound copper was removed using a Toyopearl HW-40 desalting column. The concentration of the LPMOs was determined by measuring the absorbance at 280 nm using theoretical extinction coefficients of 54,360, 46,910, 75,775, and 29,450 M^−1^ cm^−1^ for *Tr*AA9A, *Nc*AA9C, *Sc*AA10C and *Sm*AA10A, respectively. ^14^C-BMCC (specific radioactivity 2.0 × 10^6^ dpm mg^−1^) was prepared as described earlier (24). To remove possible cellulose bound metal ions the ^14^C-BMCC was incubated with 10 mM EDTA in 10 mM Tris–HCl, pH 8.0 overnight. Finally, EDTA was removed by washing with 50 mM sodium acetate (pH 5.0) using repetitive centrifugation and resuspension steps. The stock solutions of ^14^C-BMCC and LPMOs were kept in 50 mM sodium acetate (pH 5.0) at 4 °C.

### Reductant peroxidase reaction

LPMO was added to the reductant (AscA or MHQ) and the reaction was started by the addition of H_2_O_2_. The oxidation of AscA was followed by the decrease in absorbance at 265 nm using appropriate calibration curves. The oxidation of MHQ was followed by the increase in absorbance at 251 nm using the extinction coefficient of 21,450 M^−1^ cm^−1^ (67). The reactions were made in 50 mM sodium acetate (pH 5.0) at 25 °C, without stirring, in a spectrophotometer cuvette.

### Cellulose peroxygenase reaction

LPMO and the reductant (AscA or MHQ) were added to ^14^C-BMCC, and 30 s after the addition of the reductant the reaction was started by the addition of H_2_O_2_. At selected times 0.18 ml aliquots were withdrawn (from a total reaction volume of 1.35 ml) and added to 20 μl of 1.0 M NaOH to stop the reaction. Cellulose was separated by centrifugation (3 min, 10^4^ × g), and the soluble products were quantified by measuring the radioactivity in the supernatant. For zero time points aliquots were withdrawn before the addition of the reductant and H_2_O_2_. The reactions were made in 50 mM sodium acetate (pH 5.0) at 25 °C without stirring.

### Measuring concentration of LPMO active in cellulose peroxygenase reaction

LPMO (250 nM) was preincubated with reductant (50 μM AscA or 1.0 mM MHQ) and 100 μM H_2_O_2_ at 25 °C. At defined times 72 μl aliquots (from a total reaction volume of 0.5 ml) were withdrawn and added to 108 μl of a mixture containing ^14^C-BMCC (1.67 g L^−1^), AscA (1.67 mM), and H_2_O_2_ (0.83 mM), followed by incubation for 15 min. The insoluble substrate was removed by centrifugation (2 min, 10^4^ × g) and the soluble products were quantified by measuring the radioactivity in the supernatant. Under these conditions the kinetics is governed by the inactivation of LPMO and the amount of released products scales linearly with the concentration of active LPMO. Calibration curves were made using different LPMO concentrations but in the absence of reductant and H_2_O_2_ in preincubation. The reactions were made in 50 mM sodium acetate (pH 5.0) at 25 °C without stirring.

## Data availability

All data are available within the article and its Supporting Information File and from the corresponding author upon reasonable request.

## Supporting information

This article contains supporting information (supplementary Figs. S1–S11) (24).

## Conflict of interest

The authors declare no competing interests with the contents of this article.
