# Supplementary material for: The “life-span” of lytic polysaccharide monooxygenases (LPMOs) correlates to the number of turnovers in the reductant peroxidase reaction
Source: J Biol Chem. 2023 Jul 26;299(9):105094. doi: 10.1016/j.jbc.2023.105094 (PMC10458328; doi:10.1016/j.jbc.2023.105094)
Supplement: Supporting information [file mmc1.docx]

Supporting information

**The “life-span” of lytic polysaccharide monooxygenases (LPMOs) correlates to the number of turnovers in the reductant peroxidase reaction**

Silja Kuusk^†^, Vincent G.H. Eijsink^§^, Priit Väljamäe^†1^

*^†^Institute of Molecular and Cell Biology, University of Tartu, Estonia.*

*^§^Faculty of Chemistry, Biotechnology and Food Science, NMBU - Norwegian University of Life Sciences, Norway.*

| TABLE OF CONTENTS |  |
| --- | --- |
|  | Page |
| Supplementary Figures |  |
| Figure S1. Progress curves and the maximum amounts of soluble products released for H_2_O_2_-driven oxidation of cellulose by *Sc*AA10C. | 2 |
| Figure S2. Characteristic progress curves for the oxidation of ascorbate in experiments with and without added H_2_O_2_. | 3 |
| Figure S3. Dependency of initial rates of ascorbate oxidation on the concentration of ascorbate in experiments without added H_2_O_2_. | 4 |
| Figure S4. Progress curves for the H_2_O_2_-driven oxidation of ascorbate by LPMOs. | 5 |
| Figure S5. Michaelis-Menten curves of the oxidation of ascorbate by LPMOs. | 6 |
| Figure S6. Apparent Michaelis-Menten parameters for the ascorbate peroxidase reaction determined at different [AscA] and [H_2_O_2_]. | 7 |
| Figure S7. Progress curves of the H_2_O_2_-driven oxidation of ascorbate by LPMOs at different concentrations. | 8 |
| Figure S8. Inactivation of *Tr*AA9A in H_2_O_2_-driven oxidation of methyl-hydroquinone (MHQ). | 9 |
| Figure S9. Progress curves of the H_2_O_2_-driven oxidation of methyl-hydroquinone by LPMOs. | 10 |
| Figure S10. Inactivation of LPMOs during the reductant peroxidase reaction is reflected in loss of activity in the cellulose peroxygenase reaction. | 11 |
| Figure S11. Relationships between kinetic parameters for the ascorbate peroxidase reaction and *n*_max_ for four different LPMOs. | 12 |
| Supplementary References | 12 |

SUPPLEMENTARY FIGURES


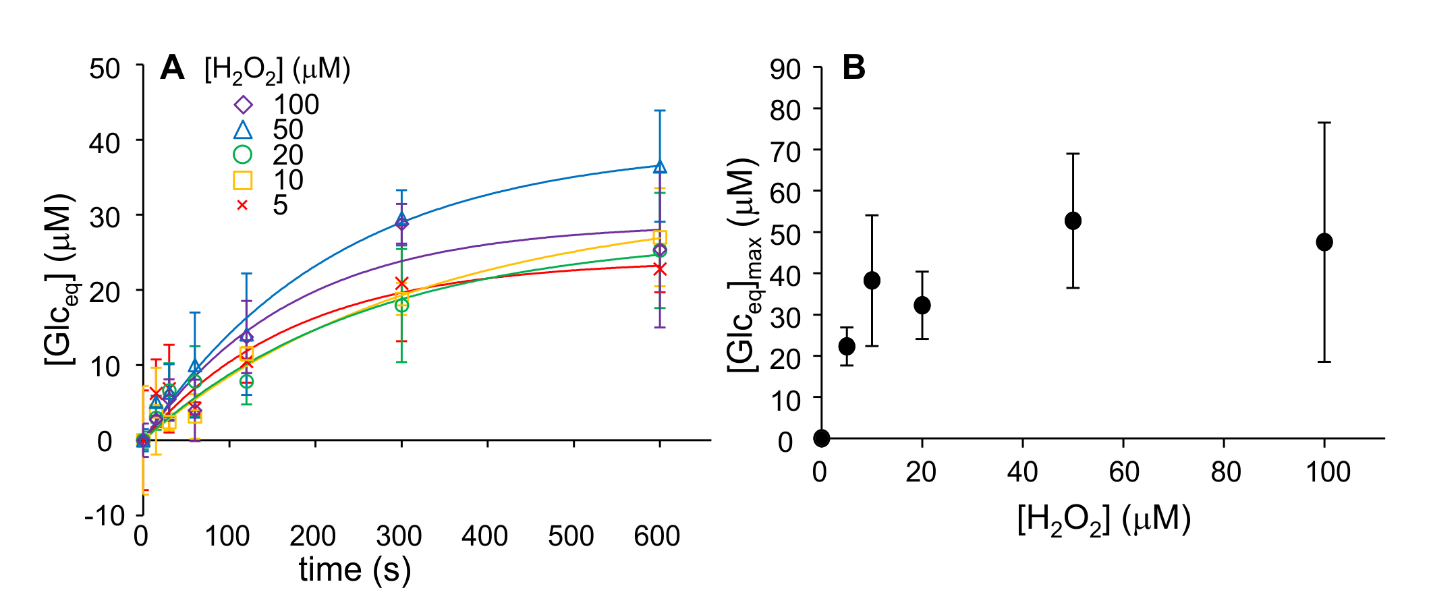


**Figure S1.** **Progress curves and the maximum amounts of soluble products released for H_2_O_2_-driven oxidation of cellulose by *Sc*AA10C.** All reactions were made in sodium acetate (50 mM, pH 5.0) at 25 ºC. The concentration of AscA was 1.0 mM and that of *Sc*AA10C was 0.02 µM. (*A*) Progress curves for the release of soluble products (in glucose equivalents, Glc_eq_) from BMCC (1.5 g L^-1^). The concentration of H_2_O_2_ is indicated in the plot. Shown are average values ± SD (*n* = 3, independent experiments). Solid lines show non-linear regression of the data according to equation 1. (*B*) Dependency of the maximum amount of soluble products ([Glc_eq_]_max_) on the concentration of H_2_O_2_. The [Glc_eq_]_max_ values were found using non-linear regression analysis of the progress curves (Fig. 1A and panel A of this figure) according to equation 1. Shown are average values ± SD (*n* = 6, independent experiments) from the experiments made using 1.0 and 1.5 g L^-1^ BMCC.


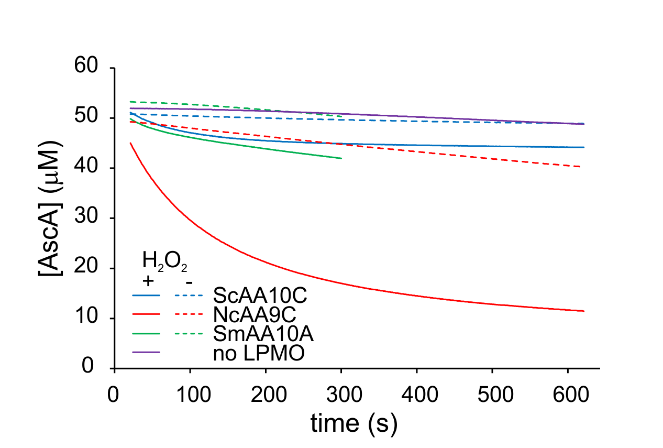


**Figure S2. Characteristic progress curves for the oxidation of ascorbate in experiments with (+) and without (-) added H_2_O_2_.** All reactions were made in sodium acetate (50 mM, pH 5.0) at 25 ºC. The concentration of AscA was 50 µM and that of H_2_O_2_ was 100 µM. The enzyme concentration was 0.25 µM for *Nc*AA9C and *Sc*AA10C, and 0.5 µM for *Sm*AA10A. The purple solid line shows the oxidation of AscA in the experiment without LPMO but with added H_2_O_2_ (LPMO independent oxidation). All progress curves for H_2_O_2_-driven oxidation of AscA by LPMOs shown in this study (Fig. 2A and Fig. S4) were compensated for oxidation of AscA in reactions without added H_2_O_2_ (dashed lines; oxidase activity of the LPMO). Traces show average values (for *Sc*AA10C *n* = 3, and for other LPMOs *n* = 2, independent experiments). SD are not shown for clarity.


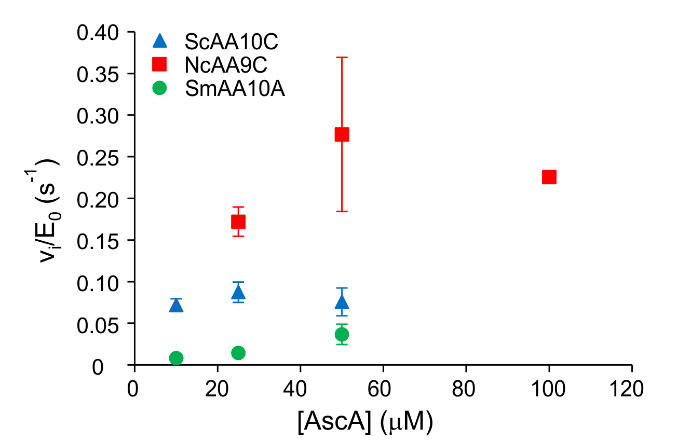


**Figure S3. Dependency of initial rates of ascorbate oxidation on the concentration of ascorbate in experiments without added H_2_O_2_.** All reactions were made in sodium acetate (50 mM, pH 5.0) at 25 ºC. Initial rates are divided by the concentration of LPMO in the experiment which was 0.25 µM for *Nc*AA9C and *Sc*AA10C, and 0.5 µM for *Sm*AA10A. These rates include also the rate of AscA oxidation in the absence of LPMO. Shown are average values and ± SD (for *Sc*AA10C *n* = 3, and for other LPMOs *n* = 2, independent experiments).


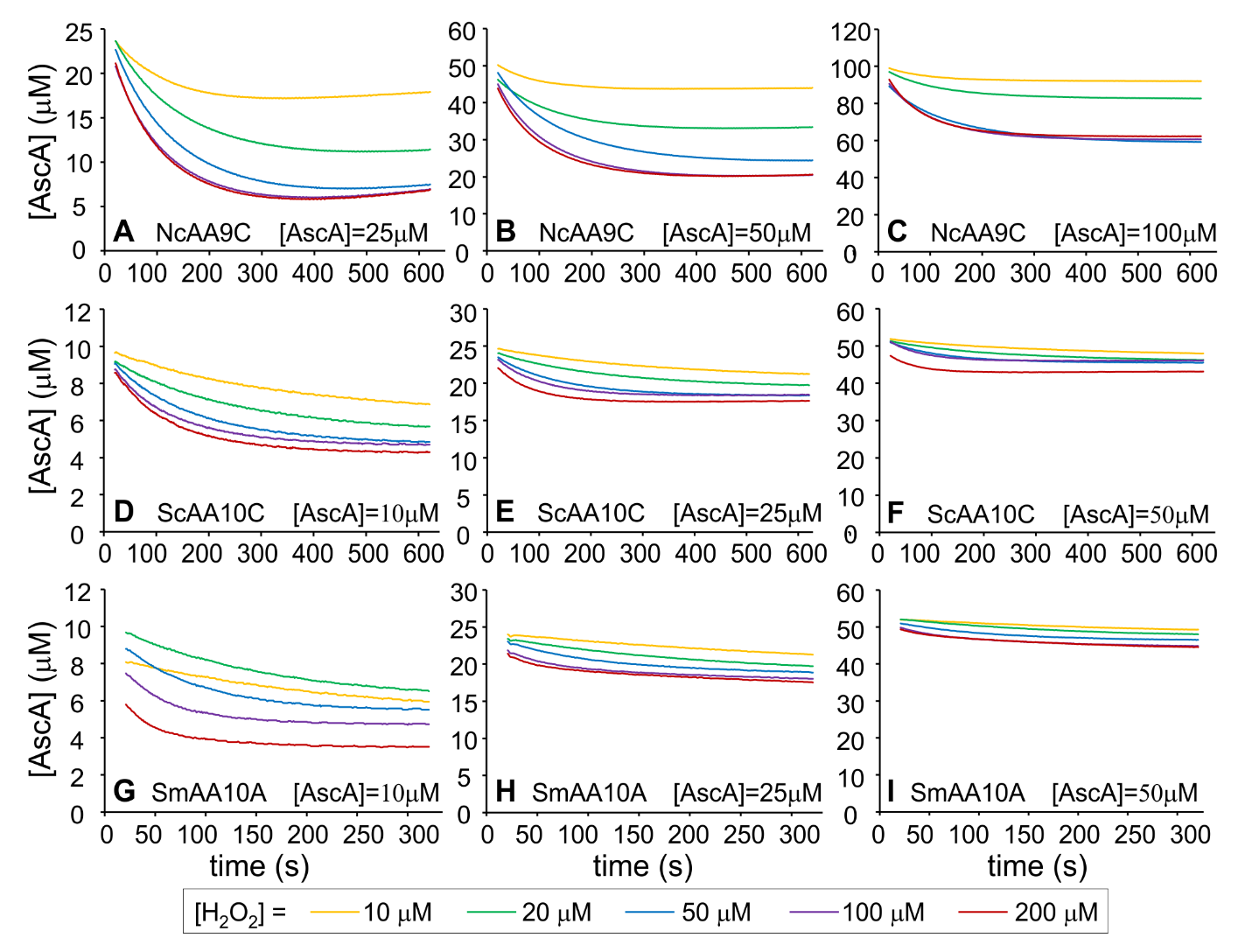


**Figure S4. Progress curves for the H_2_O_2_-driven oxidation of ascorbate by LPMOs.** All reactions were made in sodium acetate (50 mM, pH 5.0) at 25 ºC. All progress curves were corrected for the oxidation of AscA in experiments without added H_2_O_2_ (see Fig. S2 as an example). Shown are the results with 0.25 µM *Nc*AA9C (*A-C*), 0.25 µM *Sc*AA10C (*D-F*), and 0.5 µM *Sm*AA10A (*G-I*). Concentrations of AscA and H_2_O_2_ are indicated in the plots. Traces show average values (for *Sc*AA10C *n* = 3, and for other LPMOs *n* = 2, independent experiments). SDs are not shown for clarity.


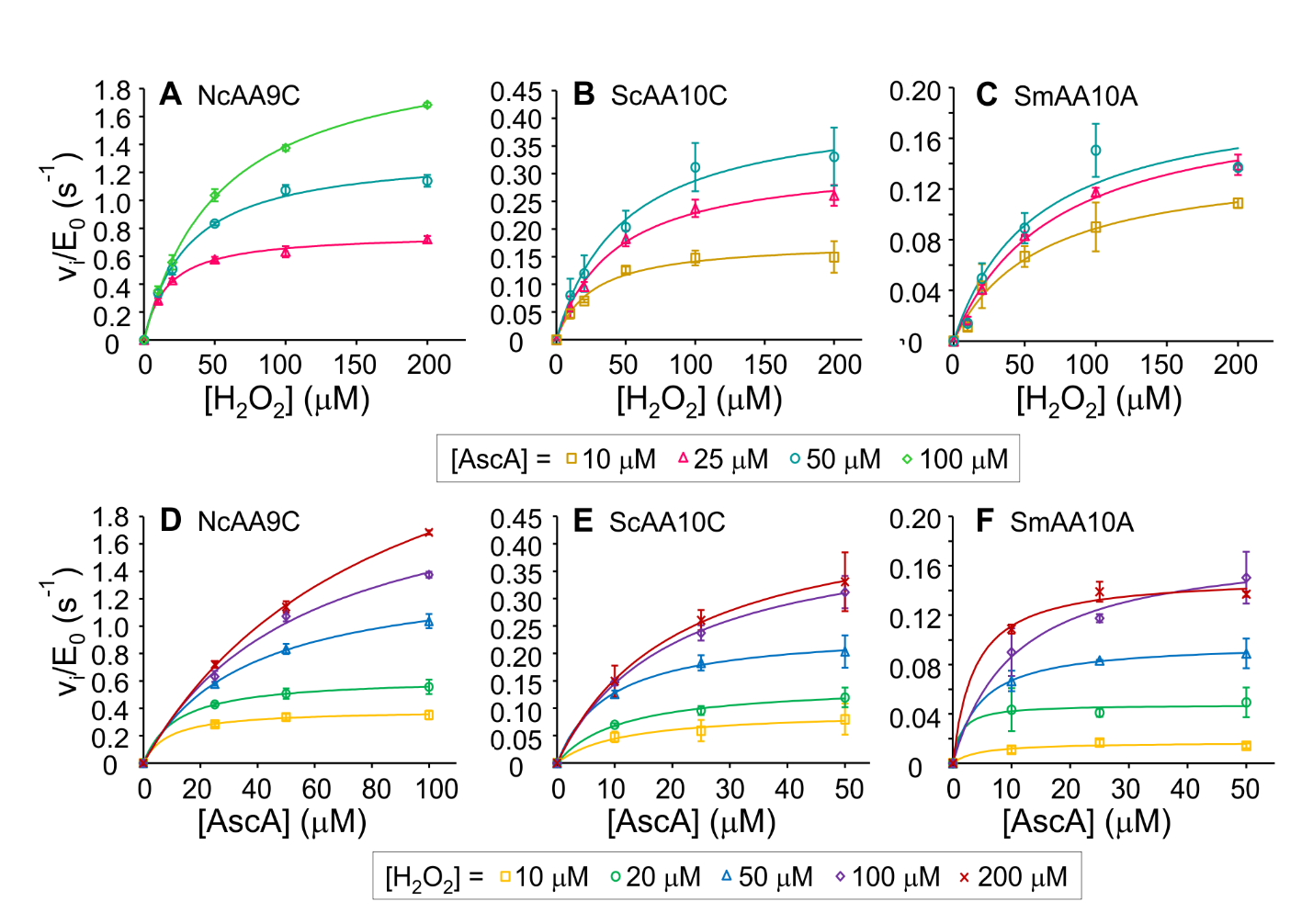


**Figure S5. Michaelis-Menten curves of the oxidation of ascorbate by LPMOs.** All reactions were made in sodium acetate (50 mM, pH 5.0) at 25 ºC. (*A-C*) Dependency of initial rates of ascorbate oxidation on the concentration of H_2_O_2_. The various concentrations of AscA differ in color, as indicated in the plots. (*D-F*) Dependency of initial rates of ascorbate oxidation on the concentration of AscA. The various concentrations of H_2_O_2_ differ in color, as indicated on the plots. Solid lines show non-linear regression of the data according to equation 2. Shown are average values ± SD (for *Sc*AA10C *n* = 3, and for other LPMOs *n* = 2, independent experiments).


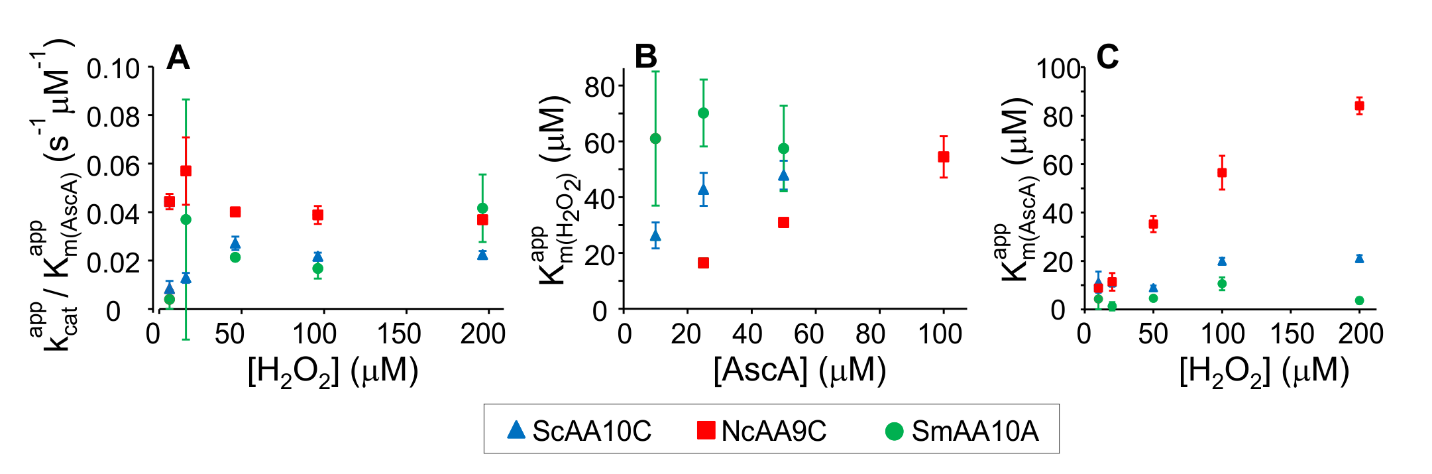


**Figure S6. Apparent Michaelis-Menten parameters for the ascorbate peroxidase reaction determined at different [AscA] and [H_2_O_2_].** All reactions were made in sodium acetate (50 mM, pH 5.0) at 25 ºC. (*A*) Dependency of the apparent *k*_cat_/*K*_M(AscA)_ on the concentration of H_2_O_2_. (*B*) Dependency of the apparent *K*_M(H2O2)_ on the concentration of AscA. (*C*) Dependency of the apparent *K*_M(AscA)_ on the concentration of H_2_O_2_. Data for the different LPMOs appear in different colors, as indicated in the plot. Shown are average values ± SD (for *Sc*AA10C *n* = 3, and for other LPMOs *n* = 2, independent experiments).


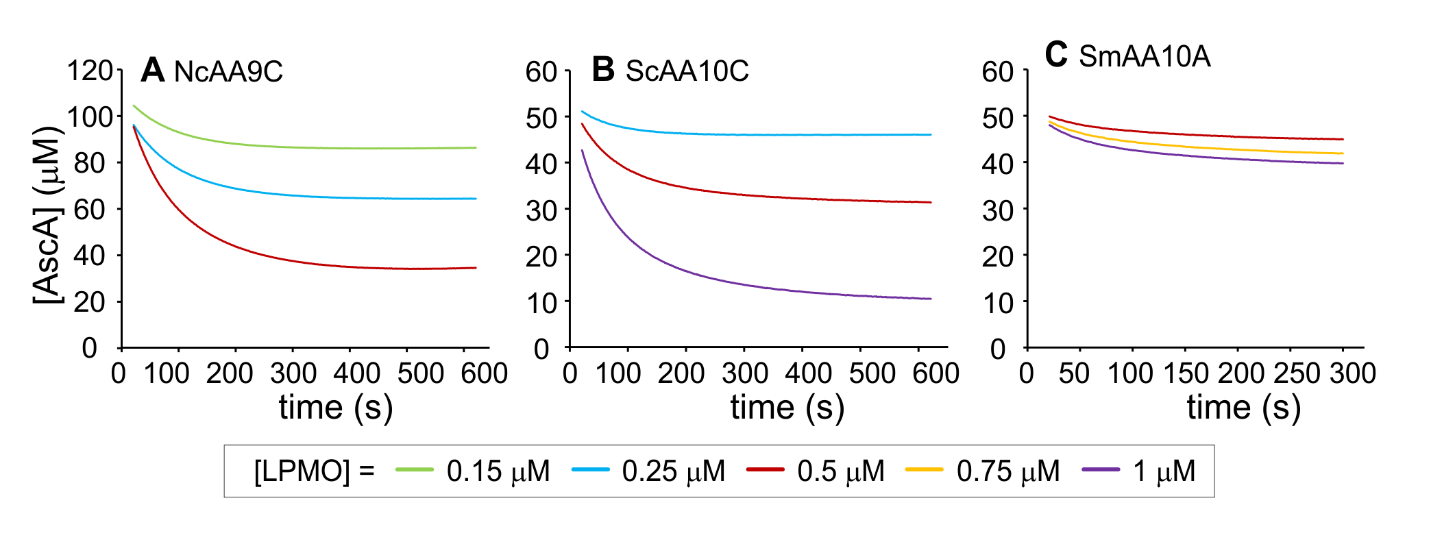


**Figure S7. Progress curves of the H_2_O_2_-driven oxidation of ascorbate by LPMOs at different concentrations.** All reactions were made in sodium acetate (50 mM, pH 5.0) at 25 ºC. All progress curves were corrected for AscA oxidation in control experiments without added H_2_O_2_ (see Fig. S2 for an example). (*A*) *Nc*AA9C with 100 µM AscA and 100 µM H_2_O_2_, (*B*) *Sc*AA10C with 50 µM AscA and 100 µM H_2_O_2_, (*C*) *Sm*AA10A with 50 µM AscA and 100 µM H_2_O_2_. The concentrations of the LPMO are indicated in the plot. Solid lines show non-linear regression of the data according to equation 4, based on average values (*n* = 2, independent experiments). SDs are not shown for clarity.


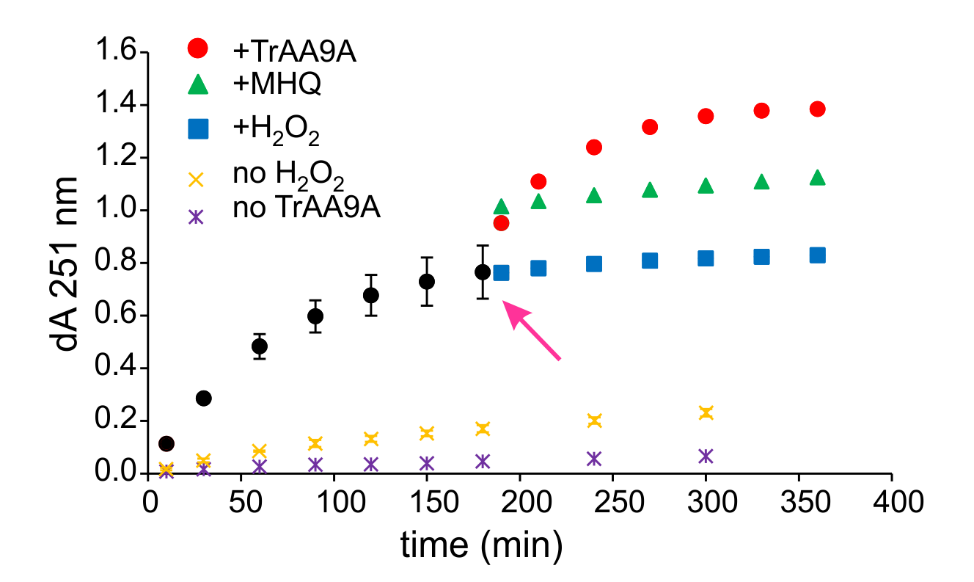


**Figure S8. Inactivation of *Tr*AA9A in H_2_O_2_-driven oxidation of methyl-hydroquinone (MHQ).** Reactions were made in sodium acetate (50 mM, pH 5.0) at 25 ºC. MHQ (1.0 mM) and H_2_O_2_ (100 µM) were incubated with 0.25 µM *Tr*AA9A. Formation of oxidized product (methyl-quinone, MQ) was followed by the increase in absorbance at 251 nm (dA 251 nm). Upon 180 min of incubation (indicated with arrowhead), the reactions were supplemented with a new portion (equal to the initial amount of the corresponding reagent) of *Tr*AA9A, MHQ or H_2_O_2_ (as indicated in the plot). Only the addition of a new portion of *Tr*AA9A caused a new burst in the rate of MHQ oxidation. The increase in absorbance upon addition of a new portion of MHQ is caused by the background absorbance by MQ present in the stock solution of MHQ. The graph also shows progress curves for experiments without added H_2_O_2_ (no H_2_O_2_) or *Tr*AA9A (no TrAA9A). The relatively high activity in the experiment without added H_2_O_2_ is caused by a small amount of H_2_O_2_ present in the MHQ stock solution (as judged by the formation of MQ product in the experiments where MHQ was incubated with horseradish peroxidase but without addition of H_2_O_2_, data not shown). We note that H_2_O_2_ present in the MHQ stock solution does not influence the measurement of the maximum amount of MHQ turned over by LPMO since these experiments were made in the excess of added H_2_O_2_.


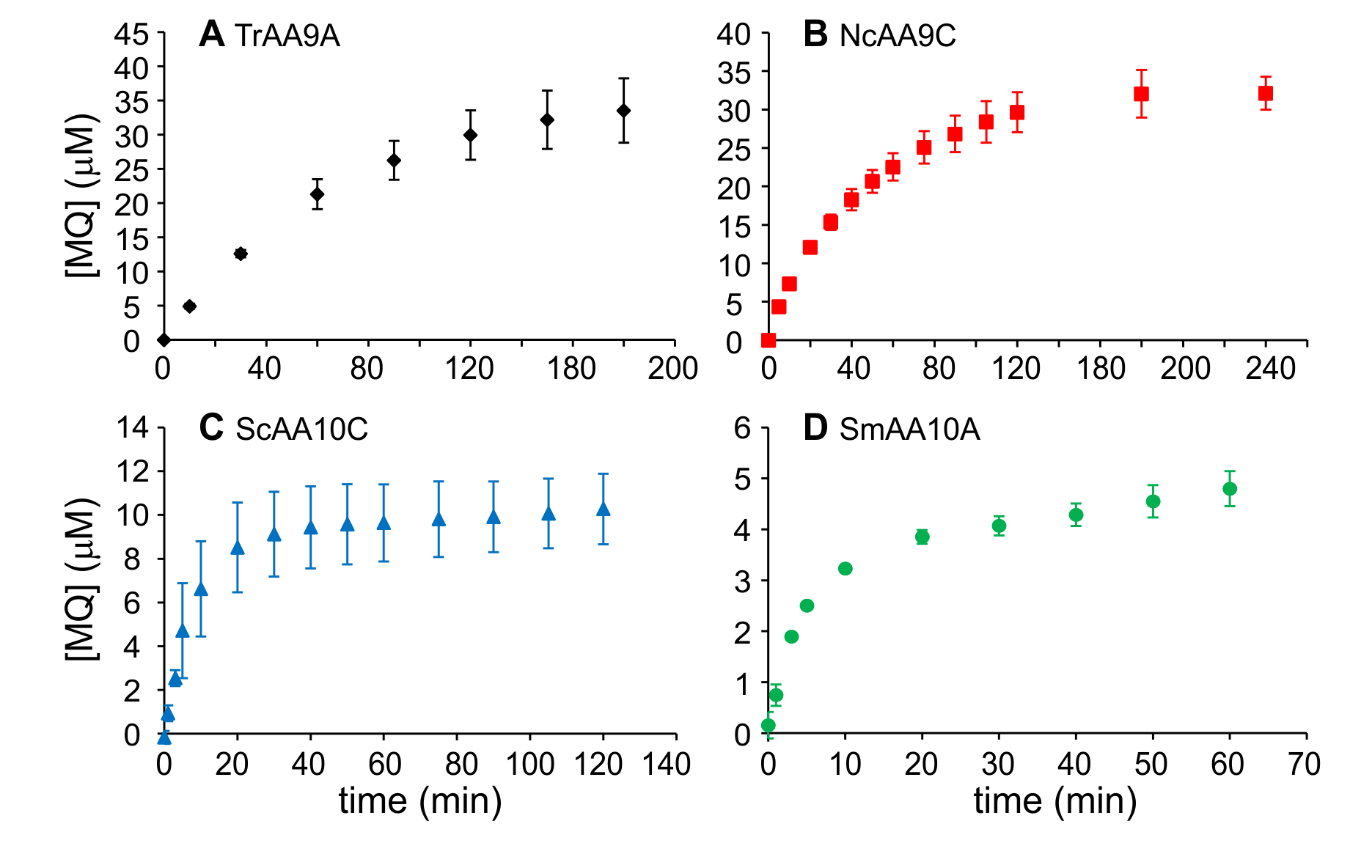


**Figure S9. Progress curves of the H_2_O_2_-driven oxidation of methyl-hydroquinone by LPMOs.** All reactions were made in sodium acetate (50 mM, pH 5.0) at 25 ºC. MHQ (1.0 mM) and H_2_O_2_ (100 µM) were incubated with 0.25 µM LPMO. The progress curves were corrected for oxidized products (methyl-quinone, MQ) formed in control experiments without LPMO. Shown are the progress curves for reactions with (*A*) *Tr*AA9A, (*B*) *Nc*AA9C, (*C*) *Sc*AA10C, and (*D*) *Sm*AA10A. Based on these results we choose 180 min incubations for *Tr*AA9A and *Nc*AA9C, and 50 min and 30 min incubations for *Sc*AA10A, and *Sm*AA10A, respectively, for the determination of maximum amount of MHQ that is turned over before inactivation of LPMO (∆[MQ]_max_).


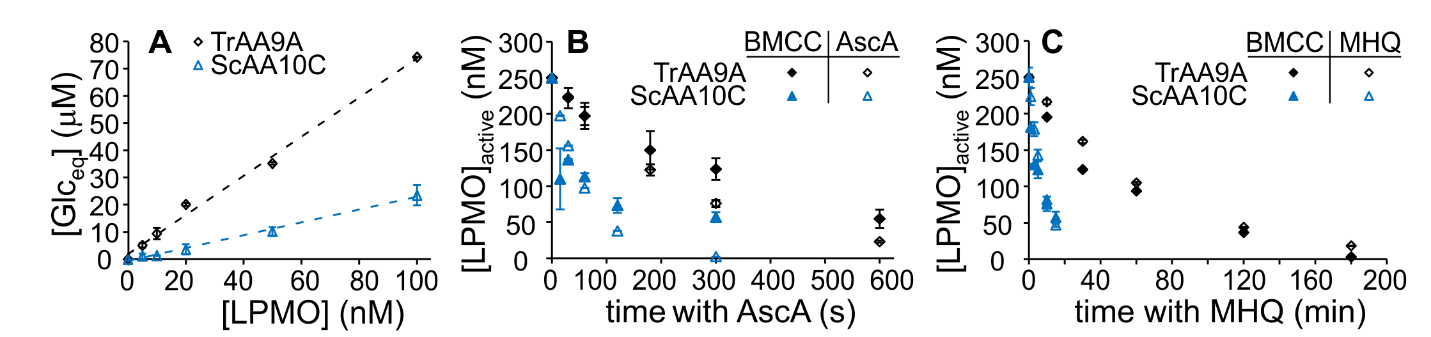


**Figure S10. Inactivation of LPMOs during the reductant peroxidase reaction is reflected in loss of activity in the cellulose peroxygenase reaction.** All reactions were made in sodium acetate (50 mM, pH 5.0) at 25 ºC. (*A*) Calibration (dose-response) curves for the cellulose peroxygenase reaction. [Glc_eq_] stands for the concentration of soluble products (in glucose equivalents) released upon incubation of BMCC (1.0 g L^-1^) in the presence of 1.0 mM AscA and 0.5 mM H_2_O_2_ for 15 min. Because of the high concentration of H_2_O_2_, the high initial activity is followed by a rapid slowdown caused by the inactivation of LPMO (inactivation driven kinetics) and the amount the product formed scales linearly with the concentration of LPMO. The figure shows average values ± SD (for *Sc*AA10C *n* = 3, and for *Tr*AA9A *n* = 2, independent experiments). Solid lines show linear regression of the data. (*B & C*) Dependency of the concentration of remaining active LPMO (250 nM starting concentration) on the time of pre-incubation with 100 µM H_2_O_2_ and 50 µM AscA (*B*) or 1.0 mM MHQ (*C*). The concentration of LPMO active in reductant peroxidase reaction was calculated according to [LPMO]_active_ = 250 nM *exp(-*k*^app^**t*). For AscA peroxidase reaction (“AscA” reactions) we used the *k*^app^ values of 0.00397 s^-1^ and 0.0157 s^-1^ for *Tr*AA9A (from Ref. 24 of the main article) and *Sc*AA10C (Fig. S4F, data with 100 µM H_2_O_2_ and 50 µM AscA), respectively. For MHQ peroxidase reaction (“MHQ” reactions) we used the *k*^app^ values of 0.0145 min^-1^ and 0.117 min^-1^ for *Tr*AA9A (Fig. S9A) and *Sc*AA10C (Fig. S9C), respectively. The concentration of active LPMO judged from cellulose peroxygenase reactions (“BMCC” reactions) was found by measuring the residual activity on cellulose in reactions containing 1.0 g L^-1^ BMCC, 1.0 mM added AscA, and 0.5 mM added H_2_O_2_, incubated for 15 min, using the calibration curves shown in panel *A*.


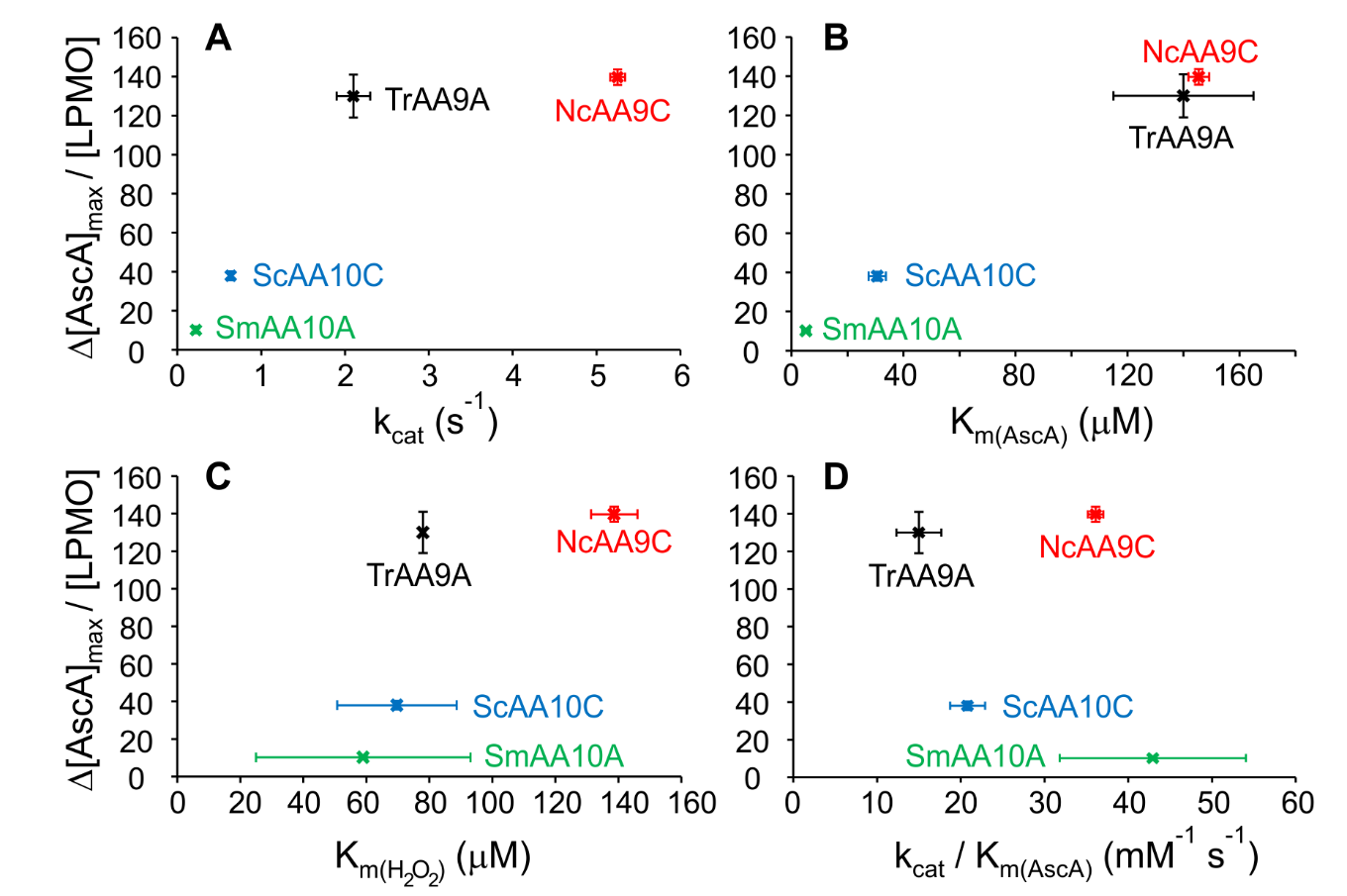


**Figure S11. Relationships between kinetic parameters for the ascorbate peroxidase reaction and *n*_max_ for four different LPMOs.** The values of true kinetic parameters and *n*_max_ (*n*_max_ = ∆[AscA]_max_/[LPMO], see eq. 5) are from Table 2 and Table 3, respectively. The graphs show the correlation between ∆[AscA]_max_/[LPMO] and (*A*) *k*_cat_, (*B*) *K*_M(AscA)_, (*C*) *K*_M(H2O2)_, and (*D*) *k*_cat_/*K*_M(AscA)_. The nature of the LPMO is indicated in the plot. The data for *Tr*AA9A are from (from Ref. 24 of the main article). Shown are average values ± SD (*n* = 2, independent experiments).
